# The effectiveness of combined resection and radiotherapy for primary pineal malignant melanoma: a systematic review

**DOI:** 10.3389/fneur.2023.1344672

**Published:** 2024-01-17

**Authors:** Jibo Zhang, Zixuan Wei, Jincao Chen

**Affiliations:** Department of Neurosurgery, Zhongnan Hospital of Wuhan University, Wuhan, China

**Keywords:** primary pineal malignant melanoma, PPMM, resection, radiotherapy, total resection

## Abstract

**Objective:**

To evaluate the effectiveness of combined resection and radiotherapy (CRAR) for the treatment of primary pineal malignant melanoma (PPMM).

**Methods:**

Relevant studies were identified through a literature search in PubMed, Embase, and Web of Science from 1899 to September 1, 2023. Then we further screened the literature according to the updated PRISMA 2020 guidelines. The article information, patient information, treatment, and survival rate were analyzed. The primary outcome measures the survival rate of CRAR compared with the overall patients and the patients without treatment. Secondary outcome measures operation methods, radiotherapy methods, and dose.

**Results:**

In total, 28 published articles were recorded. Among them, 35.71% (10/28) articles were on CRAR. The median overall survival, CRAR, and no treatment survival were 65, 88, and 12 weeks, respectively. The median overall survival of CRAR was demonstrably better than that of no treatment (*p* < 0.0001) and overall survival, even with *p* = 0.1177. Most of the operations adopted a supracerebellar infratentorial approach, and stereotactic radiation to tumor bed usually ranged between 50 and 60 Gy. Small dose and multiple fractions was the most popular radiotherapy method.

**Conclusion:**

Currently, CRAR, compared with other treatments, is more beneficial to prolonging the survival of PPMM patients. However, many more clinical cases are needed to verify it as the best treatment approach.

## Introduction

Primary malignant melanoma is an uncommon occurrence within the central nervous system, particularly in the pineal region. Primary pineal malignant melanoma (PPMM) poses a significant challenge for neurologists, akin to handling a delicate and complex situation. There is currently no definitive consensus regarding the optimal approach for its treatment. There exists a divergence of opinions among medical professionals regarding the most appropriate course of action, namely surgical intervention, radiotherapy, or chemotherapy.

According to the published literature (1), combined resection and radiotherapy (CRAR) is one of the most effective methods to treat PPMM. However, the effect of CRAR on PPMM compared with other treatment methods is still unknown. Currently, there are no guidelines on the specific operation methods, radiotherapy methods, or dose.

In this study, we aimed to evaluate the effectiveness of combined resection and radiotherapy for primary pineal malignant melanoma, with additional data from our local institution.

## Methods

This systematic review adhered to the PRISMA (Preferred Reporting Items for Systematic Reviews and Meta-Analyses) guidelines (2). Electronic database searches were performed on PubMed, Embase, and Web of Science. Search keywords included a combination of ‘Pineal’ and ‘Melanoma’. Studies were included if (1) the study was clinical research about humans and (2) the study reported the patient’s treatment and prognosis. Studies were excluded if (1) the intracranial tumors in the study were metastatic tumors or non-pineal region tumors or (2) the study was systematic/narrative review or meta-analysis without reporting new relevant cases.

We recorded the retrieved published articles. The article information, patient information, treatment, and survival rate were analyzed. The primary outcome measures the survival rate of CRAR compared with the overall patients and the patients without treatment. Secondary outcome measures operation methods, radiotherapy methods, and dose.

All analyses were performed with GraphPad Prism V.8.2.1 (GraphPad Software, Inc., La Jolla, CA, United States). Kaplan–Meier survival curves were compared using the log rank test. Results were considered to achieve statistical significance if *p* < 0.05.

## Results

**Figure 1:**
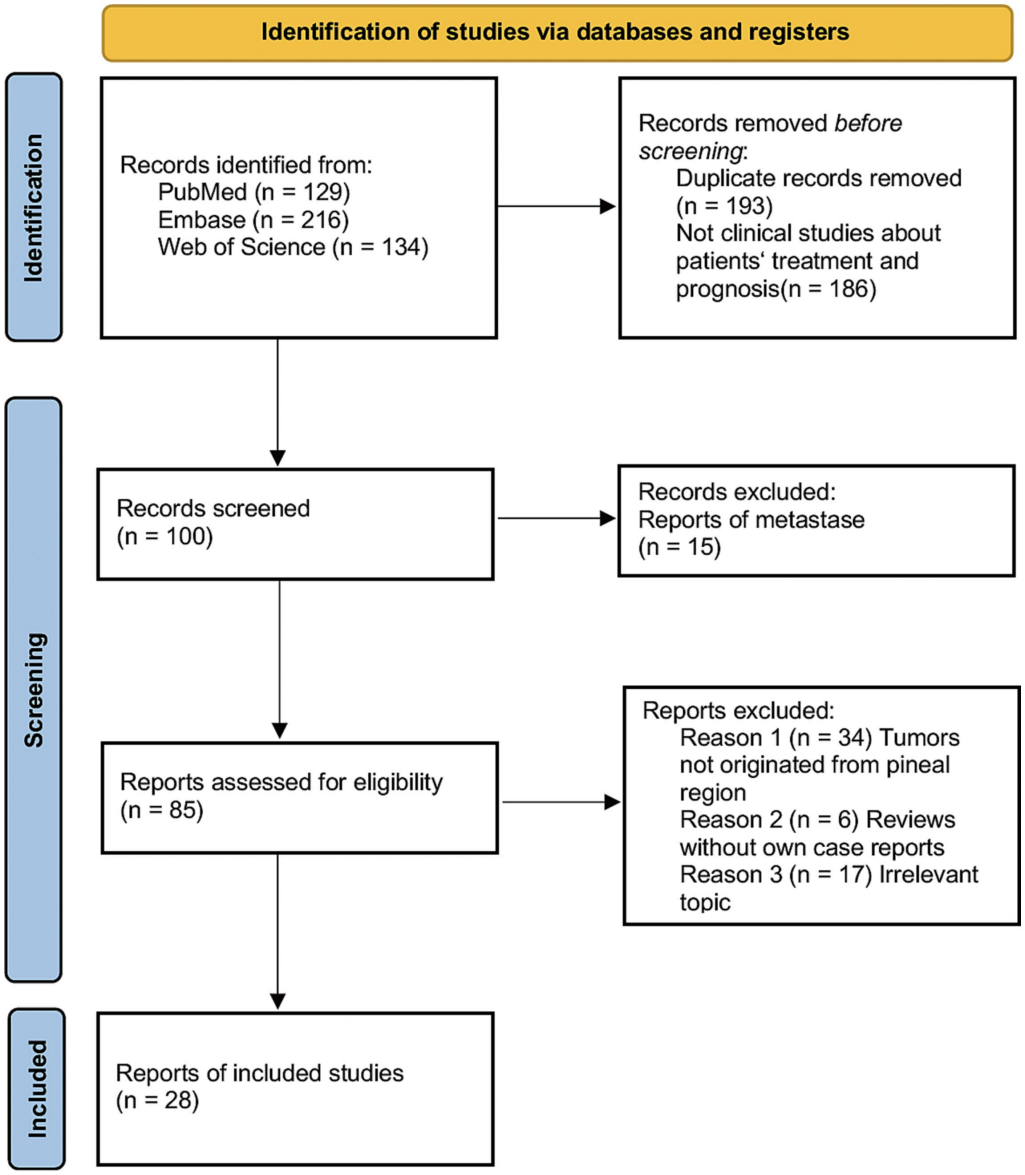
PRISMA flow chart.

**Figure 2:**
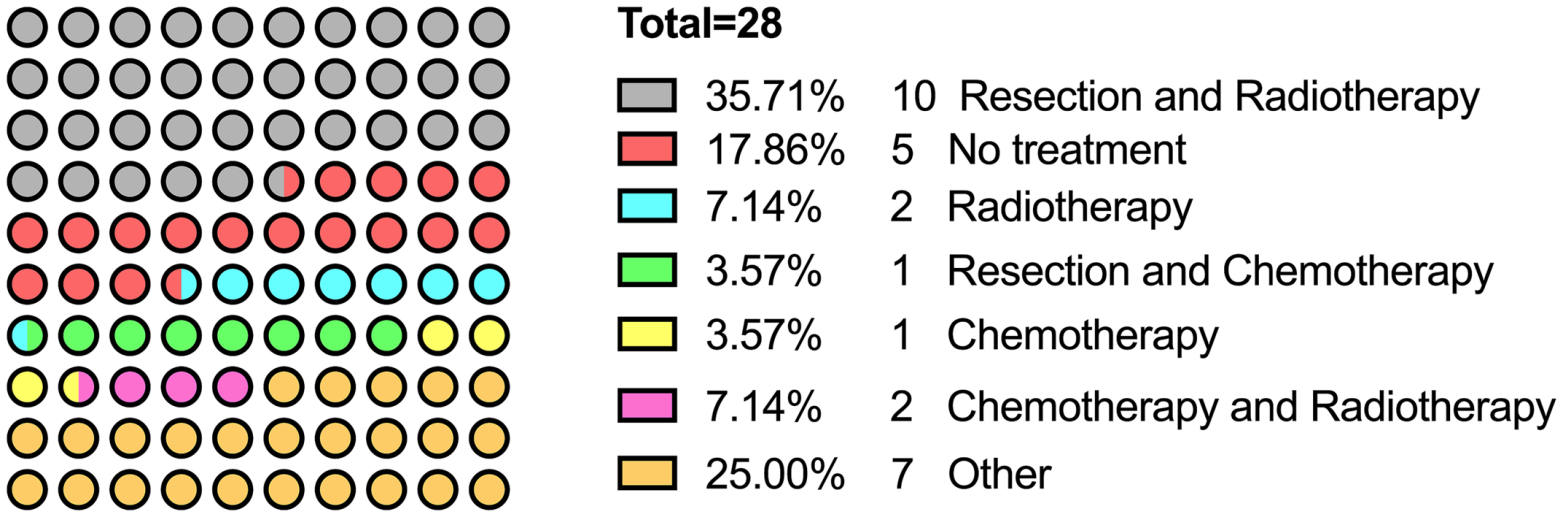
Proportion of various treatments.

The search queries yielded a total of 479 abstracts from three different databases. After removing the duplicate records and screening according to several criteria, 28 studies were finally included in the study (1, 3–29) (Table 1). Figure 1 presents the search and selection process. The coverage range was 1899–2023, most of which come from Europe and America. The average age of patients was 52.71 (range 20–77, median 52.5) years old. The proportion of men was 53.6% (15/28). Of the articles, 35.71% (10/28) articles were on CRAR and 17.86% (5/28) articles were on no treatment (Figure 2).

**Table 1:** Literature review of primary pineal melanoma.

Study	Year of publication	Country	Age (yrs)	Sex	Resection	Chemotherapy	Radiotherapy	Biopsy	VPS/EVD	Survival (wks)
Ogle (3)	1899	United States	32	F	−	−	−	−	−	13
Stoerk et al. (4)	1904	Germany	31	M	−	−	−	−	−	12
Foot et al. (5)	1931	United States	49	M	−	−	−	−	−	4
Gibson et al. (6)	1957	Canada	68	F	−	−	−	−	−	8
Enriquez et al. (7)	1973	Spain	43	M	−	−	−	−	−	37
Arlant et al. (8)	1977	United States	56	M	−	−	+	−	−	56
Carlson et al. (9)	1987	United States	77	F	−	−	−	+	+	5
Weindling et al. (10)	1988	United States	59	M	−	−	−	+	−	−
Rubino et al. (11)	1993	United States	60	M	+	−	+	−	−	>52
Yamane et al. (12)	1994	Japan	53	F	+	+	−	−	−	>280
Mitchell et al. (13)	1998	Australia	49	M	−	−	−	+	−	−
Czirják et al. (14)	2000	Hungary	48	M	−	−	−	−	−	−
Suzuki et al. (15)	2001	Japan	50	F	+	−	+	−	−	88
Bookland et al. (16)	2007	United States	20	F	−	+	+	+	+	>37
Barron et al. (17)	2007	Canada	73	F	−	−	+	−	−	69
Martin-Blondel et al. (18)	2009	France	44	M	−	+	+	−	−	52
Cedeño Diaz et al. (19)	2011	Spain	70	M	+	−	+	−	−	16
Arantes et al. (20)	2011	Portugal	54	F	+	+	+	+	+	>80
Shinshato et al. (21)	2012	Japan	49	F	+	−	+	−	−	>56
Azimi et al. (22)	2012	Iran	22	F	+	−	−	+	−	7
Park et al. (23)	2014	Korea	59	M	+	−	+	−	−	>26
Biswas et al. (24)	2015	India	45	F	+	−	+	−	−	>40
Jetschke et al. (25)	2015	Germany	57	M	−	+	−	−	−	3
Wendel et al. (1)	2018	Germany	52	M	+	−	+	−	−	65
Hajhouji et al. (26)	2019	France	52	F	+	−	+	−	−	>60
Famoso et al. (27)	2019	United States	75	F	+	−	+	−	−	>138
Zhang et al. (28)	2020	China	67	M	+	−	+	−	−	>104
Aaroe et al. (29)	2021	United States	62	M	−	+	+	+	−	−

M, Male, F, Female; VPS, Ventriculoperitoneal Shunt; EVD, External Ventricular Drain.

**Figure 3:**
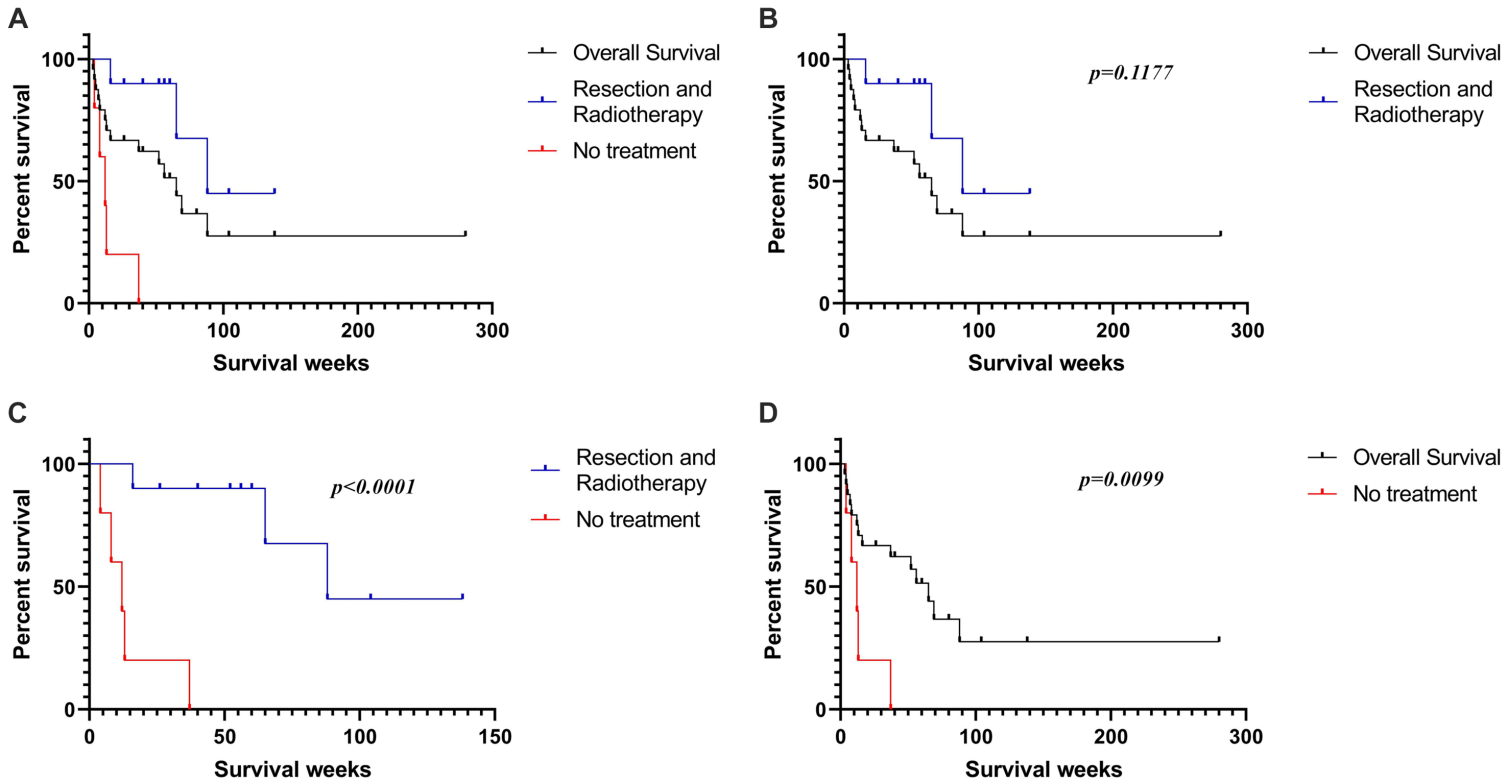
**(A)** The Kaplan-Meier survival curves of all reported PPMM patients, as well as the patients who received CRAR and those who did not receive treatment. **(B)** Patients who received CRAR showed a trend of better median overall survival than all reported PPMM patients (*p* = 0.1177). **(C)** The median overall survival of untreated patients was significantly lower than that of patients who underwent surgical resection combined with radiotherapy (*p* < 0.0001). **(D)** The median overall survival of untreated patients was significantly lower than the overall median survival of all reported PPMM patients (*p* = 0.0099).

In Figure 3, Kaplan–Meier survival curves were compared in overall, CRAR, and no treatment cases. The median overall survival of CRAR was obviously better than that of no treatment (*p* < 0.0001) and overall survival, even with *p* = 0.1177. The median overall survival was approximately 65 weeks, ranging from 3 to 280 weeks. Patients who received CRAR treatment had a median survival of 88 weeks. In the absence of treatment, the median survival was reduced to only 12 weeks.

In Table 2, we demonstrate that most of the operations adopted a supracerebellar infratentorial approach, a common approach for the treatment of pineal region tumors. As for the method of radiotherapy, whole-brain radiotherapy was most common in the past, while stereotactic radiation to tumor bed is more commonly used now. Tumor control doses usually range between 50 and 60 Gy. Small dose and multiple fractions was the most popular radiotherapy method. Local data were similar to the above treatment methods and have received good results.

**Table 2:** Literature review of operation and radiotherapy methods of primary pineal melanoma.

Study	Year of publication	Country	Age (yrs)	Sex	Operative approach	Radiotherapy methods	Radiotherapy dose	Survival (wks)
Rubino et al. (11)	1993	United States	60	M	Supracerebellar approach	Whole-brain irradiation	NA	>52
Suzuki et al. (15)	2001	Japan	50	F	Right occipital transtentorial approach	Whole-brain irradiation	50Gy	88
Cedeño Diaz et al. (19)	2011	Spain	70	M	Supracerebellar infratentorial approach	NA	NA	16
Shinshato et al. (21)	2012	Japan	49	F	Occipital transtentorial approach	Whole brain and extended local boost irradiation	Whole brain (36Gy) and extended local boost(18Gy) irradiation	>56
Park et al. (23)	2014	Korea	59	M	Occipital transtentorial approach	Whole brain and extended local boost irradiation	Whole brain (36Gy) and extended local boost(18Gy) irradiation	>26
Biswas et al. (24)	2015	India	45	F	Supracerebellar infratentorial approach	Tumor bed (pineal region) in local radiation	59.4 Gy / 33 fractions / 6½ weeks (at 1.8 Gy per fraction)	>40
Wendel et al. (1)	2018	Germany	52	M	Supracerebellar infratentorial approach	Stereotactic radiation	3 × 7 Gy for 2 successive days with CyberKnife	65
Hajhouji et al. (26)	2019	France	52	F	suboccipital infratentorial supracerebellar approach	Gamma knife radiosurgery	NA	>60
Famoso et al. (27)	2019	United States	75	F	Supracerebellar infratentorial approach	Stereotactic intensity-modulated radiation to the postoperative bed and residual gross tumor	54 Gy, delivered in 30 daily fractions of 1.8 Gy	>138
Zhang et al. (28)	2020	China	67	M	Supracerebellar infratentorial approach	Stereotactic radiation to tumor bed	54 Gy/ 30 fractions /6 weeks (5 days per week) / 1.8 Gy per fraction	>104

M, Male; F, Female; NA, Not Available.

## Discussion

The aim of the review was to evaluate the effectiveness of CRAR for PPMM. The included 28 studies reported PPMM cases, including their treatment and prognosis. Of the studies, 35.71% focused on CRAR. Our study found that CRAR has a good effect compared with the overall survival rate of patients without treatment.

Primary intracranial malignant melanoma (PIMM) accounts for only 1% of all melanoma cases (30). Due to the limitations of the blood–brain barrier on the efficacy of radiotherapy and chemotherapy, previous literature has recommended surgical resection as the main treatment for PIMM (31). Our study similarly suggests that resection plays a crucial role in treating PPMM. We should make every effort to completely remove the tumors. The supracerebellar infratentorial approach, which offers optimal tumor exposure, is considered the most favorable option. The combined use of a microscope and endoscopy technique has proven to be effective in treating pineal region tumors. The maximum extent of tumor removal can be guaranteed. In this approach, the tumor is initially removed through surgery using a microscope. Any remaining tumor is then identified and removed using endoscopy. Endoscopy is also used to observe whether the mesencephalic aqueduct is obstructed by blood clots and to exclude the occurrence of hydrocephalus after operation.

For radiotherapy, so far, stereotactic radiation to tumor bed for PPMM usually ranges between 50 and 60 Gy. Small dose and multiple fractions was the most popular radiotherapy method. Radiotherapy has been reported to increase the permeability of the blood–brain barrier and is believed to delay the recurrence of metastatic malignant melanoma (32, 33). Therefore, radiotherapy remains one of the primary treatments for intracranial malignant melanoma. Furthermore, the utilization of radioenhancers to enhance the effectiveness of radiation therapy and the advancement of stereotactic radiosurgery, specifically gamma knife radiosurgery and cyber knife radiosurgery (which can now be administered stereotactically, thereby reducing the undesired exposure of the chiasm and pituitary stalk to gamma knife and other forms of radiation therapy), have the potential to enhance the prognosis of patients (34, 35). Our results demonstrated the effectiveness of CRAR in prolonging the survival period of patients with PPMM, which also supported the potential role of radiotherapy in the treatment of PIMM. However, due to the very few cases of PPMM who only received radiotherapy, our study could not clearly define the value of radiotherapy in the treatment for PIMM.

Obviously, there are few reports about the therapeutic effect of chemotherapy, which means a clear conclusion cannot be drawn yet. Yamane et al. (12) conducted a case study on a patient who underwent resection and chemotherapy and achieved a survival time of over 280 weeks. This represents the longest reported survival data for patients with PPMM. Martin-Blondel et al. (18) reported a patient who survived for 52 weeks after undergoing chemotherapy and radiotherapy. Jetschke et al. (25) reported a case where a patient who received chemotherapy alone had a survival time of only 3 weeks. Wendel et al. (1) reported that chemotherapy administration could potentially be delayed until recurrence occurs. Regardless of the surgical procedures and additional therapies used, it is widely believed that achieving complete tumor removal is extremely important. Maybe in the future, PPMM research on cells and animals in laboratory can improve the treatment of the disease in humans. The tumor cells are treated with radiotherapy and / or chemotherapy *in vitro*. And after the cells are injected into the pineal region of mice, they are treated with surgery and / or radiotherapy and / or chemotherapy *in vivo*.

In addition to radiotherapy and chemotherapy, emerging adjuvant therapies such as targeted therapy and immunotherapy have demonstrated clear effectiveness in managing melanoma brain metastases. Approximately 50% of melanomas have a mutation in the serine–threonine protein kinase B-RAF (BRAF) gene, leading to activation of the MAPK pathway. About 90% of BRAF mutations occur at codon 600, with BRAFV600E (substituting valine with glutamic acid) being the most common mutation (36). To date, three BRAF inhibitors have been approved for treating metastatic melanoma. The identification of BRAF mutations in PPMM is gaining more attention (1, 25). Only one case of a BRAFV600E mutation in a PPMM has been documented. In this case, the patient’s condition worsened 2 weeks after surgery and resulted in death 26 days after the operation. Nevertheless, this finding suggests that some PPMM patients may respond well to BRAF-targeted therapy (25). Immunotherapy is mainly used to treat patients with multiple melanoma brain metastases, with drugs like ipilimumab and pembrolizumab shown to be effective (37). Famoso et al. reported a case of a PPMM with positive PD-L1 expression. After partial tumor removal and radiotherapy, the patient was treated with pembrolizumab and remained recurrence-free for 138 weeks (27). This finding confirms the potential of immunotherapy in treating PPMM.

Most of the previous reports were from America or Europe. Our previous report is the first study in China (28). In fact, we also searched the Chinese database for PPMM reports, but found nothing. We do not know whether there is any possibility of different incidence rates of disease between different countries and regions. This needs more data to verify in the future.

## Conclusion

Currently, CRAR, compared with other treatments, is more beneficial to prolonging the survival of PPMM patients. However, many more clinical cases are needed to verify it as the best treatment approach.

## Data availability statement

The original contributions presented in the study are included in the article/supplementary material, further inquiries can be directed to the corresponding author.

## Author contributions

JZ: Writing – original draft, Data curation, Formal analysis, Methodology. ZW: Writing – original draft, Data curation, Formal analysis. JC: Writing – review & editing, Funding acquisition.
